# Up-regulation of *DGAT1* in cancer tissues and tumor-infiltrating macrophages influenced survival of patients with gastric cancer

**DOI:** 10.1186/s12885-021-07976-5

**Published:** 2021-03-09

**Authors:** Ping He, Shihuan Cheng, Feng Hu, Zhanchuan Ma, Yan Xia

**Affiliations:** 1 Department of Gastroenterology, The First Hospital of Jilin University, Changchun, 130021 Jilin China; 2 Department of Rehabilitation, The First Hospital of Jilin University, Changchun, Jilin 130021 People’s Republic of China; 3 Department of Hepatology and Gastroenterology, The Second Part of First Hospital of Jilin University, Changchun, China; 4 Central Laboratory, The First Hospital of Jilin University, Changchun, Jilin China; 5 Key Laboratory of Organ Regeneration and Transplantation, Ministry of Education, Changchun, 130021 Jilin China

**Keywords:** Diacylglycerol-acyltransferase 1, Gastric cancer, Prognosis of patients, Cancer treatment

## Abstract

**Background:**

Diacylglycerol-acyltransferase 1 (*DGAT1*) plays an important role in the energy storage and is involved in cancer progression. A growing number of evidences showed that elevated expression of *DGAT1* in cancer tissue indicated a poor outcome in cancer patients. However, the relationship between *DGAT1* and gastric cancer is still unclear. Thus, Transcriptomic analysis and in vitro experiments were performed to investigate the role of *DGAT1* in gastric cancer, as well as the potential therapy target in gastric cancer treatment.

**Methods:**

We screened the public cancer datasets to identify the expression and function of *DGAT1* in gastric cancer and tumor infiltrating lymphocytes. Then we testified the *DGAT1* expression and function after sodium oleate treatment in AGS and MKN45 cell line. Finally, we analyzed ration of apoptosis, necrosis in gastric cancer cells by using flow cytometry after administration of *DGAT1* inhibitor.

**Results:**

Our results showed a highly expression of *DGAT1* in gastric cancer tissues (*n* = 5, *p* = 0.0004), and tumor-infiltrating macrophages with elevated *DGAT1* expression is associated with poor overall survival in gastric cancer patients. In addition, gastric cell lines AGS (*n* = 3, *p* < 0.05) and MKN45 (*n* = 3, *p* < 0.01) expressed higher level of *DGAT1* than human gastric mucosal epithelial cell line GES-1. Administration of *DGAT1* inhibitor effectively suppressed functional factors expression and induced cell death in MKN45.

**Conclusion:**

The findings of this research provide an in-depth insight into the potential role and influences involved in *DGAT1* in the gastric cancer patients. And higher expression of *DGAT1* leads to lower overall survival (OS) rate in patients with poorly differentiated gastric cancer. Our findings suggest a potential role for *DGAT1* in the gastric cancer progression and inhibiting *DGAT1* might be a promising strategy in gastric cancer treatment.

**Supplementary Information:**

The online version contains supplementary material available at 10.1186/s12885-021-07976-5.

## Background

Cancer is a leading cause of death to human beings [1]. Gastric cancer is responsible for more than 900,000 deaths in the past year, rapidly growing number of new cases and deaths make it the third leading cause of cancer death in the world [1, 2]. Also, data from The World Health Organization (WHO) demonstrated that a growing number of new cases of gastric cancer occur in developing countries [3]. Regionally, people in East Asia, East Europe, and South America have higher gastric cancer incidence [4]. And it remains a tough work to cure gastric cancer in worldwide, primarily because most patients present with advanced disease [5]. Clinical data showed that advanced stage of gastric patients have poor five-year survival rate [5]. Evidence suggests that early diagnosis is critical for gastric cancer treatment and improving sufferers’ survival [6]. However, despite its extensive clinical studies, early diagnosis of gastric cancer still cannot meet the requirement due to the lack of appropriate biomarkers [6].

Generally, adipose tissue is the primary site to syntheses fatty acid in animals [7]. For example, it was reported that brown adipose tissues produced angiopoietin-like 4 play an important role in controlling lipoprotein metabolism, deficiency of which leaded to improvement of triglyceride clearance without loss of body weight in mice [8]. However, evidences showed that tumor tissue can also produce abundant fatty acid to facilitate lung tumorigenesis by upregulating long-chain family member 3 level and augmenting cellular ATP in KRAS mutant mouse model [9]. Further, an in vitro study demonstrated that accumulation of lipid droplets in colon cancer induced mitochondrial respiration indirectly promotes tumor growth and metastasis by expanding tumor-associated macrophages [10]. These studies suggest that fatty acid and the related metabolic signaling pathway might have the potential to determine the local milieu and hence tumor growth.

Triacylglycerol (TAG) is the major energy source stored in human adipose tissue [8]. Diacylglycerol-acyltransferase 1(*DGAT1*) is responsible for synthesis of TAG after utilizing two substrates diacylglycerol and fatty-acyl CoA with respect to lipid metabolism [11]. *DGAT1* is a transmembrane protein that can be found in the endoplasmic reticulum of several types of cells. Data from several studies suggest that suppressed *DGAT1* is a promising strategy to inhibit prostate cancer cell growth and, therefore, targeting *DGAT1* might be conducive for clinical gastric cancer treatment [12, 13]. It has been previously reported that the elevated level of reactive oxygen species is facilitated to cancer progression and metastasis, and gastric cancer cell proliferation and migration could be suppressed after administration of inhibitor to block the reactive oxygen species production [14–16]. Another study demonstrated that accumulation of the nicotinamide adenine dinucleotide phosphate oxidase 2 (NOX2) activates Akt, Stat3, and IκBα signaling pathways in two gastric cancer cells. Restrained NOX2 expression decreases cyclin D1 expression and leads to cell growth arrest [16]. However, much uncertainty still exists about the relationship between *DGAT1* and the reactive oxygen species in gastric cancer progression. The objectives of this research are to determine whether *DGAT1* functionally influence gastric cancer progression and prognosis of patients, and uncover the clinic meaning of *DGAT1*.

## Methods

### ONCOMINE database

ONCOMINE database (www.oncomine.org) is a convenient online cancer database that unifies high-throughput cancer profiling data across a large volume of cancer types, subtypes, and experiments [17]. In our study, transcriptional expressions of *DGAT1* between different types of cancer tissues and their corresponding adjacent normal control samples were obtained from ONCOMINE database. Difference of transcriptional expression was compared by students’ t-test. Cut-off of *p* value and fold change were as following: *p* value: 1E-4, fold change: 2, gene rank: 10%, data type: mRNA. The used datasets were listed as follow: Superficial Bladder Cancer, Sanchez-Carbayo Bladder 2; Myeloma, Zhan Myeloma 3; Pancreatic Adenocarcinoma, Iacobuzio-Donahue Pancreas 2; Ovarian Serous Cystadenocarcinoma, TCGA Ovarian; Papillary Renal Cell Carcinoma, Yusenko Renal; Gastric cancer, DErrico Gastric.

### UALCAN

UALCAN (http://ualcan.path.uab.edu) is an open access database for analyzing cancer OMICS data from the The Cancer Genome Atlas (TCGA) [18]. Target genes can be tested for biomarkers identification in various cancers. It also provides platform to evaluate gene expression in molecular subtypes of different cancers and corresponding adjacent normal tissues. We obtained expression data pertaining to gastric cancer, and we limited our search to stomach adenocarcinoma, Difference of transcriptional expression was compared by students’ t test and *p* < 0.05 was considered as statically significant.

### cBioPortal

The cBioPortal for Cancer Genomics (http://cbioportal.org) is a comprehensive database provides resource for investigating and analyzing multidimensional cancer genomics data from clinical and basic researches [19]. We obtained expression data pertaining to gastric cancer, and we limited our search to stomach adenocarcinoma. In this study, we evaluated aberrant expression of *DGAT1* in patients with gastric cancer based on TCGA database, difference of transcriptional expression was compared by students’ t test and when a *p* value < 0.05, the difference was considered statically significant. Other detailed parameters could be found in the additional file.

### Survival analysis

Kaplan-Meier plotter (http://kmplot.com/analysis/) is a useful online database for the evaluation of prognostic value of target genes in cancer patients [20]. We obtained expression data pertaining to gastric cancer, and we limited our search to stomach adenocarcinoma. Patient samples with poorly differentiated gastric cancer were divided into two groups according to the median expression of the target gene, and overall survival (OS), first progression (FP), and post progression survival (PPS) of patients were plotted to evaluate value of target genes between high expression group and low expression group, with the hazard ratio (HR) with 95% confidence intervals (CIs) and log rank *p* value.

### Systematic analysis of immune infiltration

TIMER (https://cistrome.shinyapps.io/timer/) is a comprehensive resource for systematic analysis of immune infiltrating cells across diverse cancer types. TIMER web server allows users to input target genes to conveniently access the tumor immunological, clinical, and genomic features [21]. In this study, we obtained expression data pertaining to gastric cancer, and we limited our search to stomach adenocarcinoma. And we evaluated correlation between *DGAT1* expression and tumor-infiltrating immune cells via “gene” modules. And “survival” module was used to explore the clinical relevance of tumor immune subsets. “SCNA” module provides the comparison of tumor infiltration levels among tumors with different somatic copy number alterations for target genes. The gene expression level was displayed with log2 RSEM. The hazard ratio (HR) and 95% CI of the clinical relevance of one or more tumor immune subsets was assessed using the Cox’s proportional hazards model, with split percentage of patients was set as 50%, and survival time was set as 120 months.

### Sample collection

The study protocols and consent forms were approved by The Ethics Review Board of First Hospital of Jilin University (number 2019–070). All participants were given written informed consent. Cancer tissue and corresponding adjacent mucosa were collected from 5 gastric cancer patients.

### Cell culture and drug treatment

Human gastric cell lines (MKN45, cat. ZQ0457; AGS, cat. Zq0240) and human gastric mucosal epithelial cell line (GES-1, cat. ZQ0905) acquired from Shanghai Zhong Qiao Xin Zhou Biotechnology Co.,Ltd. (http://www.zqxzbio.com/Index/index.html) in 2020. All cell lines were tested by PCR and found to be mycoplasma negative. All cell lines were authenticated by profiling of STRs (Short Tandem Repeats) analysis.

Gastric cancer cell line AGS and MKN45 were cultured at 37 °C in a 5% CO_2_ atmosphere in RPMI 1640 medium supplemented with 10% fetal bovine serum (Gibco, USA) and penicillin/streptomycin (100 U/ml/ 100 μg/ml, Yeasen, ShangHai, CN). Human gastric mucosal epithelial cell GES-1 was cultured at 37 °C in a 5% CO_2_ atmosphere in DMEM medium supplemented with 10% fetal bovine serum (Gibco, USA) and penicillin/streptomycin (100 U/ml/ 100 μg/ml, Yeasen, ShangHai, CN).

Sodium oleate was purchased from Shanghai Aladdin (https://www.aladdin-e.com/), dissolved with heated double distilled water upon received, and then filtered with 0.22 μM filter. Stock solution (200 mM) stored aliquots at − 80 °C under sterile conditions. *DGAT1* inhibitor A922500 was purchased from the selleck, China (https://www.selleck.cn/), dissolved with dimethylsulfoxide upon received. Stock solution (100 mM) stored aliquots at − 80 °C under sterile conditions.

5 × 10^5^ indicated cells were cultured in 6 well plates and treated with sodium oleate (200 μM) and/or A922500 (100 μM) for 12 h

### RNA extraction and quantitative real-time PCR

Total RNA was extracted from cells stimulated with sodium oleate and/or A922500 for 12 h with Trizol (Invitrogen) regent. Then cDNA was synthesized using TransScript First-Strand cDNA Synthesis SuperMix (TransGen Biotech). qRT-PCR was performed with an ABI StepOnePlus system (Applied Biosystems) with a SYBR Green Kit (TransGen Biotech). The expression level was normalized against the β-actin. Relative mRNA expression was calculated using the 2 − ΔΔCT method. The primer sequence sets used for *DGAT1*, NOX2, IDO, and β-actin was listed as follows:

**Table:** 

Primer name	Primer sequence
*h/m-actin*	F: 5′ TTCAACACCCCAGCCATG 3′ R: 5′ CCTCGTAGATGGGCACAGT 3’
*h-DGAT1*	F: 5’ TATTGCGGCCAATGTCTTTGC 3 R: 5’CACTGGAGTGATAGACTCAACCA 3’
*h-NOX2*	F: 5’ CACAGGCCTGAAACAAAAGA 3′ R: 5′ GCTTCAGGTCCACAGAGGAA 3’
*h-IDO*	F:5’ GCCCTTCAAGTGTTTCACCAA 3′ R:5′ GCCTTTCCAGCCAGACAAATAT 3’

### Cell apoptosis analysis

The MKN45 cells were treated with sodium oleate and/or A922500 for 24 h. Then cells were collected and suspended in 100 μl incubation buffer, stained with Annexin V-fluorescein isothiocyanate (FITC, 5 μl) and propidium iodide (PI, 10 μl) for 15 min at room temperature, then 400 μl Binding Buffer was added to cells and cell apoptosis was then analyzed using an Ariall flow cytometer (BD Biosciences). Data were evaluated using FlowJo software (Version 10; FlowJo).

### Statistical analysis

Significance was determined with the independent-samples Student’s t-test analysis. Where indicated, statistical analysis was performed on Prism 7.0 (GraphPad Software), and *p* < 0.05 was considered statistically significant. Data are representative of three independent experiments with similar results. Quantification of signal was shown in bar graphs and error bars represent mean ± SD.

## Results

### Abnormally elevated expression of *DGAT1* in patients with gastric cancer

**Fig. 1:**
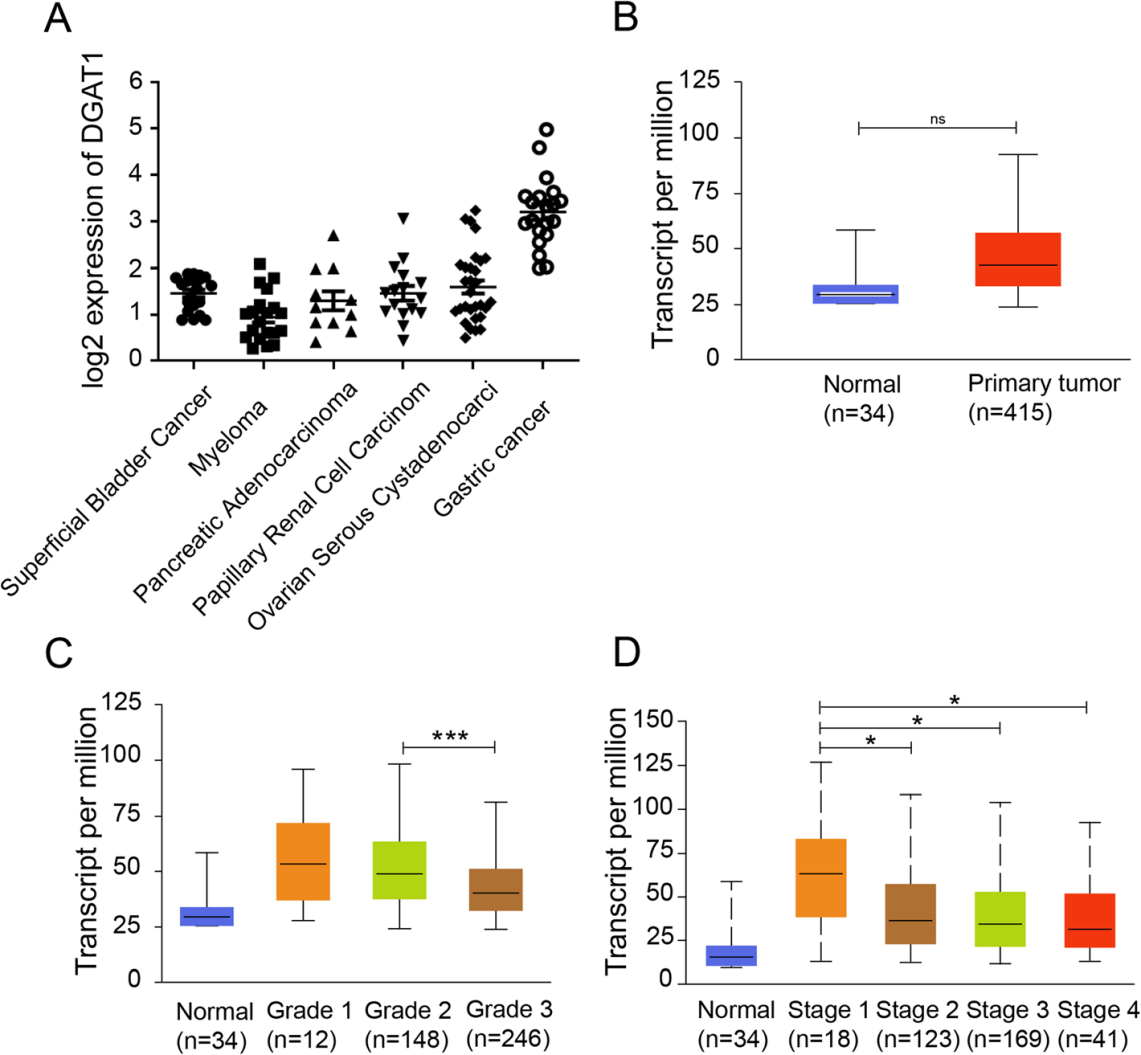
The expression of *DGAT1* was elevated in gastric cancer patients and then decreased with cancer progression. **a** The expression of *DGAT1* in pan-cancer (Superficial Bladder Cancer, *n* = 20; Myeloma, *n* = 20; Pancreatic Adenocarcinoma, *n* = 11; Papillary Renal Cell Carcinom, *n* = 16; Ovarian Serous Cystadenocarcinoma, *n* = 29; Gastric cancer, *n* = 20). **b** Comparison of *DGAT1* expression between normal tissues and primary tumors in stomach adenocarcinoma patients (*p* = 0.25054). Comparison of *DGAT1* expression upon different tumor grades (**c**) or individual cancer stages (**d**). *p < 0.05; ***p < 0.001. Tumor grades details: Grade 1: well differentiated (low grade); Grade 2: moderately differentiated (intermediate grade); Grade 3: poorly differentiated (high grade); Grade 4: undifferentiated (high grade). Data in (**a**) were collected from the Oncomine database, Data in (**b**-**d**) were collected from the UALCAN database

We initially investigated expression of *DGAT1* in pan-cancer of patients by collecting data from the Oncomine database. *DGAT1* level elevated in multiple cancer, such as bladder cancer, myeloma, pancreatic adenocarcinoma, papillary renal cell carcinoma, ovarian cancer, and gastric cancer (Fig. 1a). Among which, *DGAT1* was highly expressed in patients with gastric cancer. Interestingly, the expression of *DGAT1* had no significant difference between normal tissues and primary tumors in stomach adenocarcinoma patients (Fig. 1b). Next, we analyzed *DGAT1* level in cancer tissues upon tumor grades or individual cancer stages, and the results showed that during the deterioration of diseases, *DGAT1* decreased in transcript level (Fig. 1c, d). Collectively, *DGAT1* was highly expressed in gastric cancer tissues, and significantly decreased with the deterioration of diseases.

### Up-regulation of *DGAT1* associated with poor prognosis in patients with gastric cancer

**Fig. 2:**
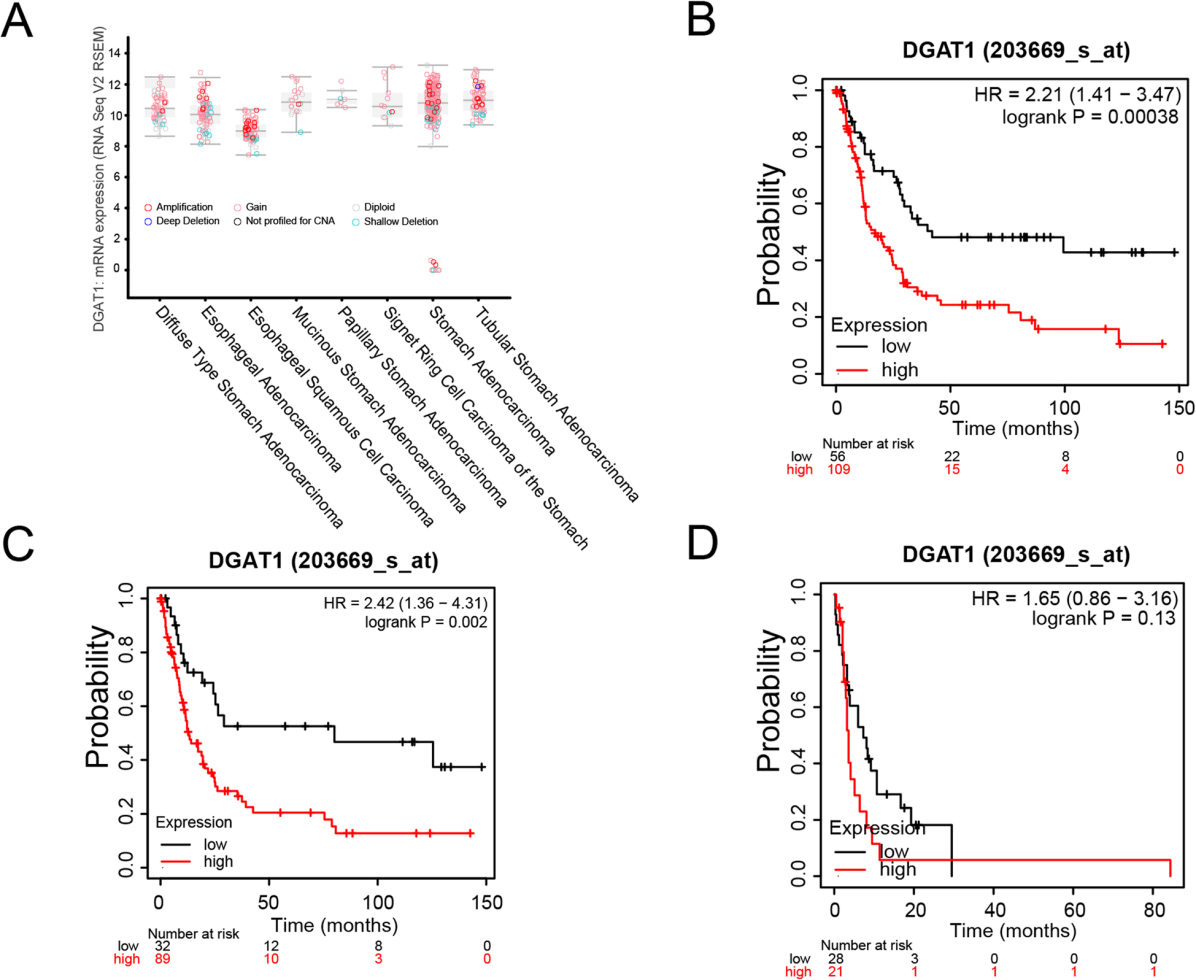
Elevated *DGAT1* expression in different types of gastric cancer indicated a worse outcome in patients. **a** Aberrant *DGAT1* expression in different gastric cancer types (Diffuse type stomach adenocarcinoma, *n* = 56; Esophageal adenocarcinoma, *n* = 85; Esophageal squamous cell carcinoma, *n* = 78; Mucinous stomach adenocarcinoma, *n* = 19; Papillary stomach adenocarcinoma, *n* = 7; Signet ring cell carcinoma of the stomach, *n* = 13; Stomach adenocarcinoma, *n* = 204; Tubular stomach adenocarcinoma, *n* = 71). Kaplan-meier plots of gastric cancer patients based on overall survival (**b**), first progression (**c**) and post progression survival (**d**). Data in (**a**) were collected from the cBioPortal database. Data in (**b**-**d**) were collected from the Kaplan-Meier Plotter database (OS, *n* = 165; FP, *n* = 121; PPS, *n* = 49)

*DGAT1* is a crucial gene involved in the conversion of diacylglycerol and fatty acyl-CoA to triacylglycerol. Deletion and aberrant amplification of *DGAT1* can be found in different types of the gastric cancer patients (Fig. 2a). To investigate the influence of altered expression of *DGAT1* to patients with gastric cancer, we compared survival time of patients depended on *DGAT1* expression level. We performed survival analysis using Kaplan-meier plots and the results showed that higher expression of *DGAT1* leads to lower overall survival (OS) rate in patients with poorly differentiated gastric cancer (Fig. 2b). Unexpectedly, survival curve analysis based on first progression analysis showed that elevated *DGAT1* expression in cancer tissues has no impact on the survival time of the indicated patients (Fig. 2c). In addition, analysis based on post progression survival showed that survival curve of two groups based on *DGAT1* expression had no significant difference (Fig. 2d). Collectively, increased *DGAT1* expression can be detected in all types of gastric cancer patients, and high level of *DGAT1* indicated a poor prognosis in gastric cancer patients.

### GO and KEGG pathway enrichment analyses of *DGAT1*

*DGAT1* is a metabolism related enzyme involved in multiple biological processes. To systematically understand role of *DGAT1* in cancer progression, we performed GO functional enrichments and KEGG pathway analysis by using the KOBAS online analysis database (http://kobas.cbi.pku.edu.cn/kobas3), with a *p*-value of < 0.05 were obtained. The results were shown in Table 1 and Table 2 (Supplementary file), *DGAT1* was mainly enriched in o-acyltransferase activity, transferase activity, transferring acyl groups, and triglyceride biosynthetic process. While the most significantly enriched pathways of the *DGAT1* were Acyl chain remodeling of DAG and TAG, triglyceride biosynthesis and metabolism, and Fat digestion and absorption. These results indicated that *DGAT1* play a key crucial role in lipid metabolism.

### *DGAT1*-expressing tumor-associated macrophage were associated with poor OS in patients with gastric cancer

**Fig. 3:**
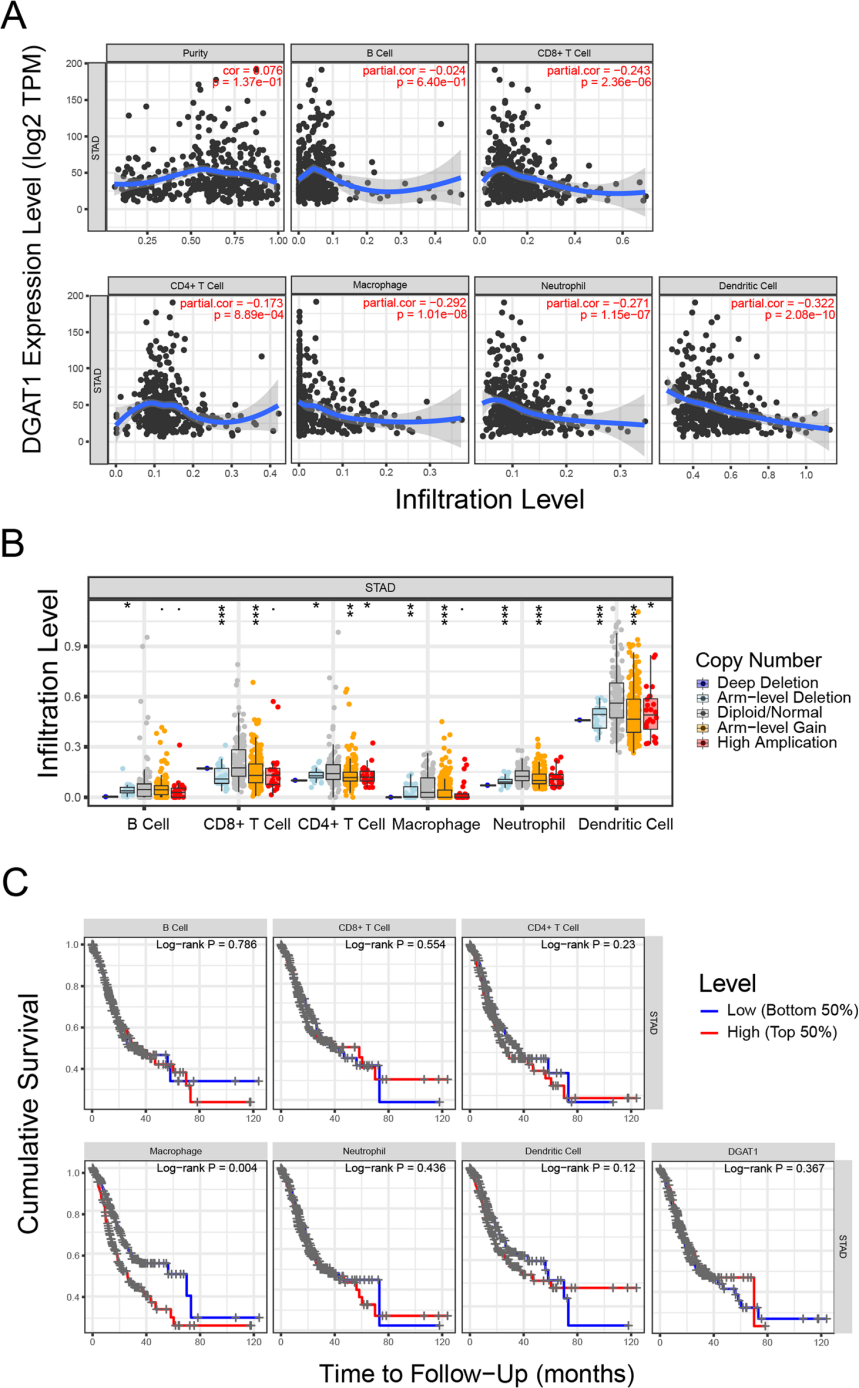
Aberrantly elevated expression of *DGAT1* in tumor-infiltrating macrophages were correlated with poor prognosis in gastric cancer patients. **a** *DGAT1* expression was significantly negatively correlated with the levels of infiltrating CD8^+^ T cells, CD4^+^ T cells, macrophages, neutrophils, and dendritic cells in STAD. **b** Comparison of tumor infiltration levels in gastric cancer with different somatic copy number alterations for *DGAT1*. **c** Increased *DGAT1* expression in tumor-infiltrating macrophages were correlated with reduced overall survival in gastric cancer patients. Data in (**a**-**c**) were collected from the TIMER database

*DGAT1* can be expressed by multiple types of cells and participate in the regulation of energy metabolism. We analyzed *DGAT1* levels in tumor-infiltrating immune cells in patients with gastric cancer. And the results revealed that mRNA expression and DNA copy number variation of *DGAT1* was increased in immune cells of gastric cancer tissue other than immune cells in normal tissues (Fig. 3a, b). Next, we explored the clinical relevance of *DGAT1* and several key tumor-infiltrating immune cell subsets under a multivariable Cox proportional hazard model. Interestingly, abnormal elevated *DGAT1* in myeloid cells, especially macrophage, was significantly associated with reduced overall survival in patients with gastric cancer (Fig. 3c), indicated that *DGAT1* could modulate the property of some immune cells and influence the prognosis of patients in an indirect way.

### *DGAT1* increased level of reactive oxygen related genes in MKN45 cells

**Fig. 4:**
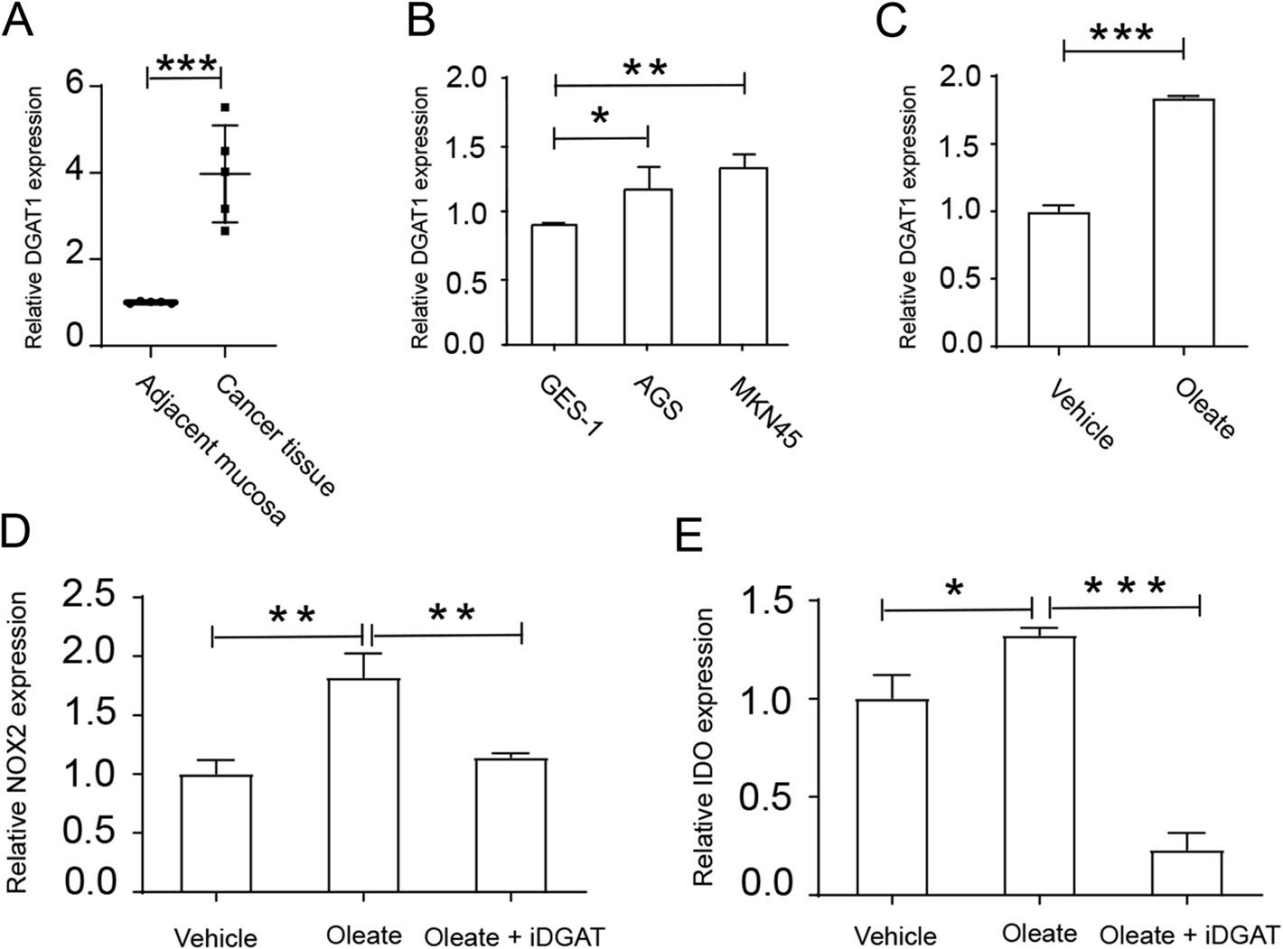
Blocking *DGAT1* pathway impaired reactive oxygen species expression in gastric cancer cell lines. **a** Gastric cancer tissues expressed higher level of *DGAT1* (*n* = 5, *p* = 0.0004). 5 × 10^5^ GES-1, AGS or MKN45 cells were cultured in 6 well plates and/or treated with sodium oleate (200 μM) and/or A922500 (100 μM) for 12 h. Then cells were collected and mRNAs were isolated for further studies. qPCR analysis of *DGAT1* (**b**) in gastric cancer cell lines, or in MKN45 after sodium oleate treatment (**c**), or NOX2 (**d**) and IDO (**e**) in MKN45 after sodium oleate and/or iDGAT treatment. *P < 0.05; **P < 0.01; ***P < 0.001. Data are representative of three independent experiments with similar results. Quantification of signal was shown in bar graphs and error bars represent mean ± SD

As high level of *DGAT1* in gastric cancer patients indicates a poor outcome with respect to overall survival, we firstly analyzed *DGAT1* expression level in cancer tissue from gastric cancer patients. Results showed that DGAT1 level was increased in the cancer tissues (Fig. 4a). Next, we investigated DGAT1 level in several gastric cancer cell lines, and human gastric mucosal epithelial cell line GES-1 was used as normal control. As shown in Fig. 4b, *DGAT1* expression was significantly higher in gastric cancer cell lines AGS and MKN45. Next, we wonder if block *DGAT1* could inhibit tumor cell growth and metabolism. As shown in Fig. 4c, oleate sodium treatment increased *DGAT1* expression in gastric cancer cell line MKN45. Surprisingly, A922500 treatment also inhibited expression of NOX2 and indoleamine 2,3-dioxygenase (IDO) in MKN45 (Fig. 4d, e), two genes correlated with proliferation and migration of gastric tumor cells [15, 16]. Collectively, oleate sodium elevated *DGAT1* expression in gastric cancer cell line MKN45 and *DGAT1* blockade induced cell apoptosis in vitro.

### *DGAT1* inhibition induced gastric cancer cell line MKN45 apoptosis

**Fig. 5:**
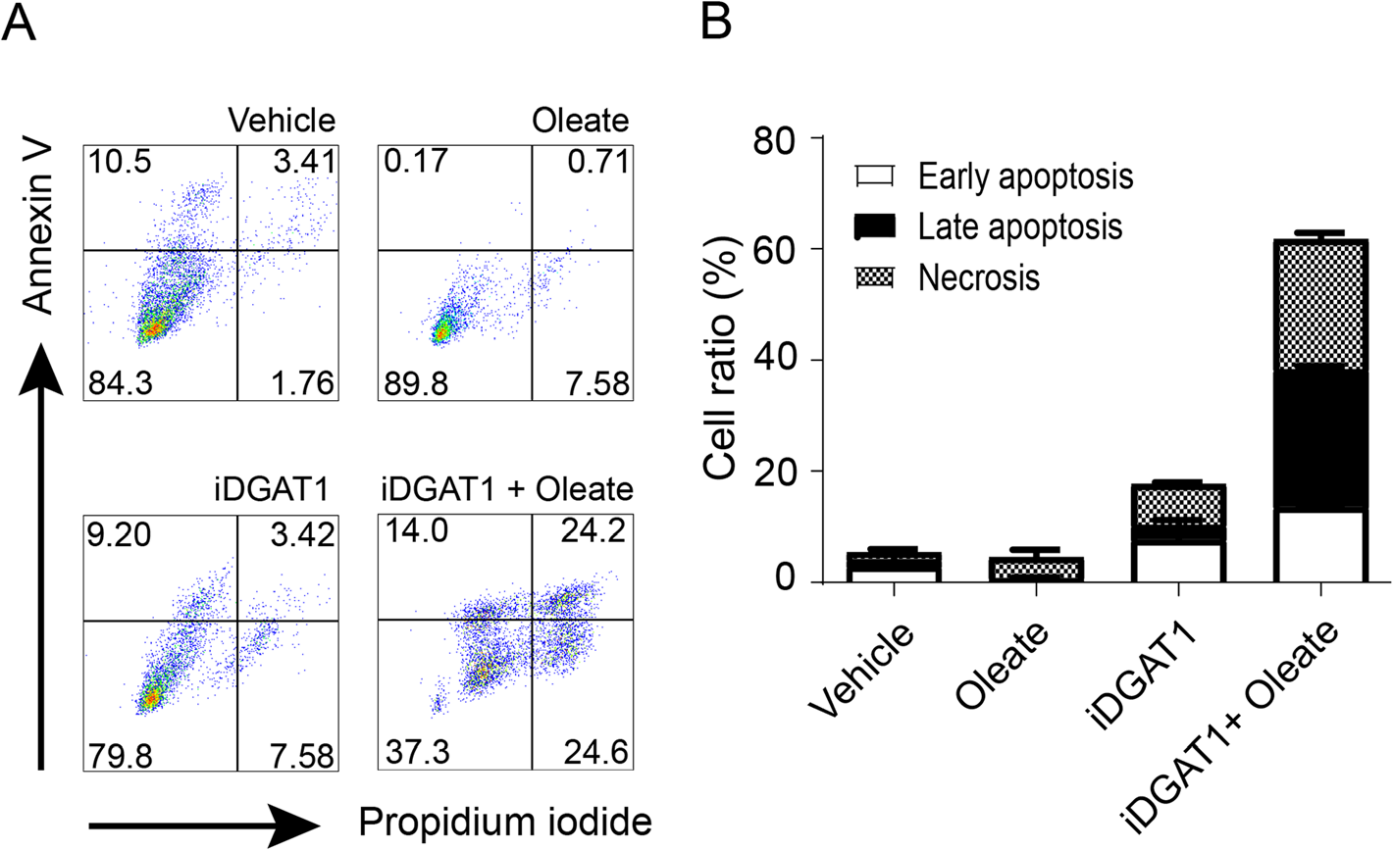
Blocking *DGAT1* lead to MKN45 cells apoptosis in vitro. 5 × 10^5^ MKN45 cells were cultured in 6 well plates and treated with sodium oleate (200 μM) and/or A922500 (100 μM) for 24 h. Then cells were collected and stained with indicated reagents for apoptosis detection. Cell apoptosis of MKN45 cells with sodium oleate and/or A922500 stimulation as examined by annexin V/PI staining (**a**, **b**). Data are representative of three independent experiments with similar results. Quantification of signal was shown in bar graphs and error bars represent mean ± SD

As block *DGAT1* effectively suppressed functional factors secretion in gastric cancer cell line, next we investigated cell viability after *DGAT1* inhibition. The results showed that addition of A922500 treatment led to increased early apoptosis and necrosis in MKN45 cells (Fig. 5a, b). To validate the effect of iDGAT on MKN45 cells, sodium oleate was added to elevate *DGAT1* level in target cells, then A922500 was added to block *DGAT1* in MKN45 cells. Remarkably, A922500 treatment in the presence of sodium oleate induced cell apoptosis and necrosis (Fig. 5a, b), which again proved that high level of *DGAT1* might facilitate the tumor growth and inhibition of increased *DGAT1* expression effectively suppressed cell expansion in vitro.

## Discussion

One of the principals aims of our study was to investigate the predicted biomarker of gastric cancer in human, and the potential target that facilitates to diagnosis and treatment gastric cancer. In this study, collected data from TCGA database reported abnormally elevated expression of *DGAT1* in several types of cancer in patients, and we observed dramatic elevated level of *DGAT1* in gastric cancer patients. Furthermore, Kaplan–Meier curves showed that aberrant expression of *DGAT1* in immune cells and tumor tissues predict a poor prognosis in gastric cancer patients, and administration of i*DGAT1* effectively inhibited reactive oxygen species expression in gastric cancer cell line, suggesting the considerable interest in *DGAT1* as a potential target for the diagnosis and treatment of gastric cancer, which indicated a potential clinical impact for controlling gastric cancer.

Previous studies have shown that *DGAT1* belongs to the membrane-bound O-acyltransferase superfamily, an acyltransferase involved in triacylglycerides synthesis and insufficient energy intake lead to triacylglycerides lipolysis [22]. During starvation, lipid droplets reduced lipotoxicity induced autophagic degradation of membranous organelles in *DGAT1* depended manner [23]. In addition, mouse with *DGAT1* deficiency are still viable but show alleviated ability to store triacylglycerols [24]. Important role of *DGAT1* in energy metabolism indicated an active role of which in rapidly proliferating cells, such as tumor cells. Indeed, we observed highly expression of *DGAT1* in several types of cancer in patients, which was especially correlated with worse prognosis in gastric cancer patients. Surprisingly, we found the elevated expression of *DGAT1* in tumor infiltrating macrophage also showed negative correlation with poor overall survival in stomach adenocarcinoma patients. Although significantly fewer studies have functionally evaluated *DGAT1* in cancer progression and metabolism, the available data reported that *DGAT1* indirectly promoted tumor growth via modulating lipid droplets formation in macrophages and enhancing suppressive function of myeloid cells to inhibit immune response in vitro [10]. Another group demonstrated that inhibition of *DGAT1* reduced prostate tumor growth [13], indicated that *DGAT1* could support tumor growth both directly and indirectly.

Myeloid cells produce large amount of reactive oxygen species to regulate inflammatory response. The phagocyte NADPH oxidase (NOX2) is mainly expressed by macrophages, neutrophils, and DCs [25], play a key role in antimicrobial immune response after activating pathogen recognition receptors expressed by those phagocytes [26]. Prior studies have shown that monocytic acute myeloid leukemia cells promotes itself survival in a NOX2 depended manner, blocking NOX2 efficiently inhibits acute myeloid leukemia cells proliferation [27, 28]. An in vitro study showed that NOX2–ROS activates multiple pathways to promotes gastric cancer cells proliferation and migration [16]. In current study, we detected sodium oleate treatment improved *DGAT1* expression in gastric cancer cell line MKN45, and NOX2 and IDO expression level also increased significantly. Meanwhile blocked *DGAT1* by the A922500 dramatically inhibited NOX2 and IDO level, and cells apoptosis detection showed an increased apoptotic ratio when compared that with control groups. Similar with NOX2, prior studies that have noted the importance of IDO in promoting tolerogenic responses under tumor inflammatory condition. In addition, IDO could be expressed by several immune regulatory cells, such as tumor-infiltrating myeloid-derived suppressor cells, that is crucial in the establishment and maintenance of cancer immune tolerance in tumor microenvironment [29]. Our study confirms that IDO is associated with tumor cells survival and inhibits IDO expression led to tumor cell apoptosis [30]. Thus, the present study raises the possibility that *DGAT1* could be a promising biomarker and potential target for the diagnosis and treatment of gastric cancer.

## Conclusions

The current study describes the crucial role of *DGAT1* in gastric cancer progression. We demonstrated a worse prognosis in gastric cancer patients with elevated *DGAT1* expression. Intervention strategies blocking *DGAT1* in patients might be clinically valuable in the future gastric cancer treatment.

## Supplementary Information

**Additional file 1.**

## Data Availability

The datasets used and/or analyzed during the current study are available from the corresponding authors on reasonable request.

## Abbreviations

*DGAT1*: Diacylglycerol-acyltransferase 1
OS: Overall survival
*NOX2*: NADPH oxidase 2
*IDO*: Indoleamine 2,3-dioxygenase
GO: Gene ontology
KEGG: Kyoto Encyclopedia of Genes and Genomes
