# Supplementary material for: Up-regulation of DGAT1 in cancer tissues and tumor-infiltrating macrophages influenced survival of patients with gastric cancer
Source: BMC Cancer. 2021 Mar 9;21:252. doi: 10.1186/s12885-021-07976-5 (PMC7941926; doi:10.1186/s12885-021-07976-5)
Supplement: Supplementary file 1 — Additional file 1. [file 12885_2021_7976_MOESM1_ESM.docx]

Table 1 GO analysis of DGAT1 associated genes with gastric cancer

| Term | Description | Count | *p*-value | adj p-values |
| --- | --- | --- | --- | --- |
| GO:0008374 | O-acyltransferase activity | 8 | 0.00016 | 0.002559 |
| GO:0016746 | transferase activity, transferring acyl groups | 3 | 0.000603 | 0.004827 |
| GO:0051235 | maintenance of location | 8 | 0.001225 | 0.006532 |
| GO:0009259 | ribonucleotide metabolic process | 19 | 0.003461 | 0.013845 |
| GO:0019432 | triglyceride biosynthetic process | 3 | 0.00536 | 0.017153 |
| GO:0043933 | protein-containing complex subunit organization | 4 | 0.007295 | 0.019454 |
| GO:1901568 | fatty acid derivative metabolic process | 7 | 0.013348 | 0.030509 |
| GO:0006793 | phosphorus metabolic process | 10 | 0.018957 | 0.036698 |
| GO:0003824 | catalytic activity | 11 | 0.020643 | 0.036699 |
| GO:0044255 | cellular lipid metabolic process | 13 | 0.023483 | 0.037281 |
| GO:0016020 | membrane | 4 | 0.025631 | 0.037283 |
| GO:1901564 | organonitrogen compound metabolic process | 8 | 0.033636 | 0.044303 |
| GO:0044237 | cellular metabolic process | 2 | 0.035996 | 0.044313 |
| GO:0010817 | regulation of hormone levels | 2 | 0.040097 | 0.045824 |
| GO:0006725 | cellular aromatic compound metabolic process | 4 | 0.043008 | 0.045874 |

Table 2 KEGG pathway analysis of DGAT1 related pathways with gastric cancer

| Term | Description | Count | *p*-value | adj p-values |
| --- | --- | --- | --- | --- |
| R-HSA-1482883 | Acyl chain remodeling of DAG and TAG | 7 | 0.000142 | 0.001864 |
| R-HSA-75109 | Triglyceride biosynthesis | 3 | 0.000248 | 0.001864 |
| R-HSA-8979227 | Triglyceride metabolism | 7 | 0.000674 | 0.002236 |
| hsa04975 | Fat digestion and absorption | 4 | 0.000745 | 0.002238 |
| hsa00561 | Glycerolipid metabolism | 6 | 0.0011 | 0.002586 |
| hsa00830 | Retinol metabolism | 12 | 0.001207 | 0.002581 |
| R-HSA-1483206 | Glycerophospholipid biosynthesis | 2 | 0.002307 | 0.004326 |
| R-HSA-1483257 | Phospholipid metabolism | 4 | 0.003781 | 0.006301 |
| R-HSA-6798695 | Neutrophil degranulation | 8 | 0.008502 | 0.012753 |
| R-HSA-556833 | Metabolism of lipids | 9 | 0.01294 | 0.017645 |
| R-HSA-168249 | Innate Immune System | 7 | 0.018531 | 0.023163 |
| hsa01100 | Metabolic pathways | 9 | 0.025453 | 0.029369 |
| R-HSA-1430728 | Metabolism | 10 | 0.036848 | 0.037221 |
| R-HSA-168256 | Immune System | 10 | 0.037221 | 0.037242 |
|  |  |  |  |  |

Detailed parameters used in databases:

| Databases | Projects | Tumor type | Sub-type details | Genes analyzed | statistical tests |
| --- | --- | --- | --- | --- | --- |
| Oncomine | DErrico et al., Eur J Cancer 2009/02 | Gastric cancer | Stomach adenocarcinoma | DGAT1 | Unpaired t test |
| UALCAN | TCGA, MET500 and CPTAC | Gastric cancer | Stomach adenocarcinoma | DGAT1 | Unpaired t test |
| TIMER | TCGA | Gastric cancer | Stomach adenocarcinoma | DGAT1 | partial correlation |
| Kaplan-Meier plotter | Andrew I ET AL., Proc Natl Acad Sci U S A. 2004/04 | Gastric cancer | not available | DGAT1 | kaplan-meier |
| cBioPortal | Wang K et al., Nat Genet. 2014/06; Miwako K et al., Nat Genet. 2014/06;  Kai W et al., Nat Genet. 2011/10;  TCGA | Gastric cancer | Stomach adenocarcinoma | DGAT1 | Unpaired t test |
